# Network pharmacology and *in vitro* experiments-based strategy to investigate the mechanisms of KangXianYiAi formula for hepatitis B virus-related hepatocellular carcinoma

**DOI:** 10.3389/fphar.2022.985084

**Published:** 2022-09-05

**Authors:** Xu Cao, Hening Chen, Zhiguo Li, Xiaoke Li, Xianzhao Yang, Qiushuo Jin, Yijun Liang, Jiaxin Zhang, Meiyue Zhou, Ningyi Zhang, Guang Chen, Hongbo Du, Xiaobin Zao, Yong’an Ye

**Affiliations:** ^1^ Dongzhimen Hospital, Beijing University of Chinese Medicine, Beijing, China; ^2^ Beijing Fengtai Hospital of Integrated Traditional and Western Medicine, Beijing, China; ^3^ Institute of Liver Diseases, Beijing University of Chinese Medicine, Beijing, China; ^4^ Key Laboratory of Chinese Internal Medicine of Ministry of Education and Beijing, Dongzhimen Hospital, Beijing University of Chinese Medicine, Beijing, China

**Keywords:** traditional Chinese medicine, HBV-related hepatocellular carcinoma, network pharmacological, bioinformatics, KangXianYiAi formula

## Abstract

The Chinese traditional medicine KangXianYiAi formula (KXYA) is used to treat hepatic disease in the clinic. Here we aim to confirm the therapeutic effects and explore the pharmacological mechanisms of KXYA on hepatitis B virus (HBV)-related hepatocellular carcinoma (HCC). We first collected and analyzed clinical data of 40 chronic hepatitis B (CHB) patients with precancerous liver lesions under KXYA treatment. Then, the cell viability, migration, cell cycle, and apoptosis of HepAD38 cells with KXYA treatment were examined. Next, we performed network pharmacological analysis based on database mining to obtain the key target pathways and genes of KXYA treatment on HBV-related HCC. We finally analyzed the expression of the key genes between normal and HBV-related HCC tissues in databases and measured the mRNA expression of the key genes in HepAD38 cells after KXYA treatment. The KXYA treatment could reduce the liver nodule size of CHB patients, suppress the proliferation and migration capabilities, and promote apoptosis of HepAD38 cells. The key pathways of KXYA on HBV-related HCC were Cancer, Hepatitis B, Viral carcinogenesis, Focal adhesion, and *PI3K-Akt* signaling, and KXYA treatment could regulate the expression of the key genes including *HNF4A, MAPK8, NR3C1, PTEN, EGFR*, and *HDAC1*. The KXYA exhibited a curative effect *via* inhibiting proliferation, migration, and promoting apoptosis of HBV-related HCC and the pharmacological mechanism was related to the regulation of the expression of *HNF4A, MAPK8, NR3C1, PTEN, EGFR,* and *HDAC1*.

## Introduction

Hepatocellular carcinoma (HCC) is the sixth most common cancer and the third leading cause of death worldwide (Sung et al., 2021). Moreover, more than 80% of HCC occurrences were related to hepatitis B virus (HBV) infection (Zhu et al., 2016), which makes the pathogenesis and treatment of HBV-related HCC still research hotspots (Couri and Pillai, 2019). During the tumorigenesis process of HBV-related HCC, HBV had oncogenic effects through its direct action and interaction with the host. Previous studies have found that the HBV gene fragments could be integrated into human chromosomes and lead to abnormal expression and function of tumor-related genes in hepatocytes (Li et al., 2014), and HBV proteins are also carcinogenic, such as HBx protein, which could promote the occurrence of HCC by stabilizing protein of Cyclin D1 (Chen et al., 2015). Moreover, HBV infection could cause chronic inflammation of the liver, which leads to repeated necrosis and regeneration of hepatocytes, increasing the accumulation of mutant genes and malignant transformation (Neuveut et al., 2010). Based on high-throughput sequencing data, the mutation rates of certain tumor-associated genes were different in HBV-related HCC and other HCC (Amaddeo et al., 2015). Therefore, exploring the molecular mechanism of HBV-related HCC will provide a more precise direction for the diagnosis and treatment of HCC.

To treat HBV-related HCC, there are two aspects needed to consider, chronic HBV infection and liver malignancy. However, the current treatment for HBV-related HCC is less than satisfactory. On the one hand, present available antiviral therapies were not yet able to achieve a complete cure for chronic hepatitis B (CHB) patients on account of the persistent existence of covalently closed circular DNA (cccDNA) of HBV (Lee and Banini, 2019). On the other hand, the existing treatments of HCC could not significantly reduce the mortality rate (GBD 2019 Diseases and Injuries Collaborators, 2020), including targeted drugs, interventional, surgical, and others. Hence, it is necessary and urgent to develop new drugs and treatments for HBV-related HCC.

Traditional Chinese medicine (TCM) has shown certain advantages for HBV-related HCC with its multi-targets and multi-pathways mode of function (Xi and Minuk, 2018). Our previous study found that a TCM formula based on Chaihu and Huangqi could improve the curative effect of CHB in a clinical trial (Li F et al., 2020). Thus, we take the KangXianYiAi (KXYA) formula mainly composed of Chaihu, Huangqi, Shanyao, and Baijiezi herbs to treat HBV-related HCC. In the current study, we first observed the therapeutic effect *via* analyzing the liver nodules change in CHB patients of a clinical case series with KXYA treatment for at least half a year. We next validated the effects of KXYA in HBV-related hepatoma cell line, HepAD38, on proliferation, migration, and apoptosis. To further explore the mechanism of KXYA on HBV-related HCC, we used the weighted gene co-expression network analysis (WGCNA) and network pharmacology, which have emerged as powerful tools to explore the connection between drugs and disease and obtain the key targets and pathways (Wang et al., 2020; Cao et al., 2021).

Our results showed that the KXYA treatment could inhibit liver nodules development and reduce serum virology indicators of CHB patients. And KXYA exerted inhibiting effects on the proliferation and migration abilities of HepAD38 cells and promoted cell apoptosis. Furthermore, we constructed an interaction network of active compound targets, disease target genes, and pathological stage-related genes for KXYA treatment on HBV-related HCC. Through the interaction network, we screened and obtained 12 key target genes. Finally, we analyzed the expression of the key target genes in normal and HBV-related HCC tissues by public data mining, and in HepAD38 cells with KXYA treatment by real-time quantitative PCR. The detailed research strategy of the study has shown in Supplementary Figure S1. To sum up, this study presented a deeper insight into the tumorigenesis mechanism of HBV-related HCC and offered more evidence and targets for the TCM treatment of HBV-related HCC.

## Materials and methods

### Preparation of KangXianYiAi formula granules and dry paste powder

The granule of the KXYA formula is composed of *Radix Bupleuri* (Chaihu in Chinese; 8 g), *Astragalus membranaceus* (Huangqi in Chinese; 16 g), *Rhizoma Dioscoreae* (Shanyao in Chinese; 15 g), and *Sinapis Semen* (Baijiezi; 3.6 g). The daily dose of KXYA granules for human adults is 0.609 g/kg, and the standard weight of adults is 70 kg. The herbs of the KXYA formula were obtained and authenticated by the Nanning Peili Pharmaceutical Co., Ltd., under the guidance of the Chinese Pharmacopeia 2015 edition (batch number: 170,407), and the specific drug granule preparation process was shown in the Supplementary Table S1. The production process of the dry paste powder of KXYA is as follows. Take the prescription amount of medicinal herbs, add 23 times of water, decoct for 2 h, filter, concentrate the filtrate into a clear paste, dry it at 70°C vacuum (−0.040–0.090 MPa) for 60 h, get the dry paste, crush it, pass through 50 mesh, mix well and pack separately. Water was purified using a Milli-Q system (Millipore, Billerica, MA, Uthe SA). In the cell experiment, the dried paste powder of KXYA formula was dissolved in dimethylsulfoxide (DMSO) to 500 mg/ml and stored at −20°C, and the KXYA solution was added to cell culture with different finaconcentrationson and use 0.1% DMSO as control. The granule of KXYA is used in human clinical case series, and the granule is brewed orally in hot water at 40°C–50°C.

### Clinical case series with KangXianYiAi formula treatment

We collected data from 40 patients with precancerous liver lesions in Dongzhimen Hospital from January 2008 to January 2020 retrospectively. Diagnostic criteria of HBV-related liver precancerous lesions: the liver cirrhosis caused by HBV infection; the size of liver nodules ≥1.0 cm under B-scan ultrasonography; the liver cancer was excluded by dynamic-enhanced MRI, dynamic-enhanced CT or contrast-enhanced ultrasound. And meeting the above three conditions at the same time can be diagnosed as HBV-related liver precancerous lesions. Reference guide for the definition of liver precancerous lesions (International Working Party, 1995; Cong et al., 2016). All the patients were treated with Entecavir (ETV) combined with d the KXYA formula for more than 6 months, and the liver nodule size was recorded by ultrasound or MRI. The basic clinic information of the patients was in Supplementary Table S2. The ethical batch number of this study is DZMEC-KY-2019-131, and the informed consent of the study has been obtained from the patients.

### Cell line

Hepatoma cell lines including HepG2, Huh7, SK-Hep1, and HepAD38 were purchased from the American Type Culture Collection (Manassas, VA, United States) and maintained in Dulbecco’s modified eagle medium (DMEM) supplemented with 10% fetal bovine serum (FBS), 100 U/ml penicillin, 100 μg/ml streptomycin and 5% CO_2_. For HepAD38, which was stably integrated with the HBV genome (Li X et al., 2020), the media were additionally supplemented with 400 μg/ml G418 sulfate (InvivoGen) (Ladner et al., 1997).

### Cell viability assay

5 × 10^3^ HepAD38 cells were seeded in 96-well plates with six duplications, after being incubated for 24 h, treated cells with 50, 100, 250, and 500 μg/ml of the gradient-concentration of KXYA. After KXYA treatment for 24, 48, and 72 h, a CCK-8 assay kit (Solarbio, CN) was carried out to assess the ability of cell growth by measuring the absorbance at the wavelength of 450 nm by the TECAN infinite M200 Multimode microplate reader (Tecan, Mechelen, Belgium). For hepatoma cell lines, 5 × 10^3^ Huh7, HepG2, and SK-Hep1 cells were seeded in 96-well plates with six duplications, after being incubated for 24 h, the cells were treated with KXYA (500 μg/ml) for 48 h. The detection method was the same as before, and the data normalized whereas taking the average number of the control groups as 1.

### Cell apoptosis detection

5 × 10^5^ HepAD38 cells were seeded in 6-well plates. After being incubated for 24 h, the cells were treated with KXYA (500 μg/ml) for 48 h. HepAD38 cells were stained with 0.1% DAPI (GlpBio, United States) for 10 min, washed with PBS, and photographed to observe the morphological changes of the cells, and we counted and calculated the ratio of apoptotic cells to total cells in three visual fields. To detect apoptosis, the phosphatidylserine level on the cell surface was estimated with Annexin V-FITC and PI apoptosis detection kit (Solarbio, CN). Apoptosis and necrosis of cells were analyzed in a flow cytometer (Beckman, United States). Results were expressed as the percentage of apoptotic or necrotic cells from total cells. The experiments were performed in triplicate and repeated 3 times.

### Cell cycle detection

To detect the cell cycle, 5 × 10^5^ HepAD38 cells were seeded in 6-well plates. After being incubated for 24 h, the cells were treated with KXYA (500 μg/ml) for 48 h. Cells were collected and fixed with 70% ethanol at 4°C overnight. After fixation, the cells were stained with propidium iodide (PI, 50 μg/ml, Solarbio, CN) for 30 min in dark. Then the samples were analyzed with a flow cytometer (Beckman, United States). The experiments were performed in triplicate and repeated 3 times.

### Cell migration assay

5 × 10^5^ HepAD38 cells were seeded in the 12-well plate. 12 h later, the cell monolayer was scratched with a sterile 10-μl pipette tip to generate a line-shaped wound. Then the cells were cultured in DMEM without FBS and treated with KXYA (500 μg/ml). After 48 h, images of the scratches were acquired with a digital camera. The scratch areas were quantified using Imagepro-plus 6.0 software (Media Cybernetics Inc., United States). The experiments were performed in triplicate and repeated 3 times, and the data normalized whereas taking the average number of the control groups as 1.

### Active compounds and putative targets in KangXianYiAi formula

We searched the components of herbs in KXYA from CNKI (https://cnki.net/) (Hu and Xiao, 2018) and TCMSP (http://tcmspw.com/tcmsp.php) (Ru et al., 2014) databases, and the conditions for determining the active ingredient are oral bioavailability (OB) ≥ 30% and drug-likeness (DL) ≥ 0.18. The corresponding compound chemical structure was acquired from the PubChem database (https://pubchem.ncbi.nlm.nih.gov) (Kim et al., 2016). Then the putative targets of active compounds were predicted with SwissTargetPrediction (http://swisstargetprediction.ch/) (Daina et al., 2019) and STITCH (http://stitch.embl.de/) (Szklarczyk et al., 2016) databases respectively, with the probability value ≥0.1 in SwissTargetPrediction database and confidence score ≥0.15 in the STITCH database.

### Retrieval of disease targets and microarray dataset about hepatitis B virus related hepatocellular carcinoma

The HBV-related HCC disease targets were gathered from GeneCards (https://genecards.org/) (Stelzer et al., 2016), NCBI Gene (https://ncbi.nlm.nih.gov/gene/) (Brown et al., 2015), OMIM (https://omim.org) (Amberger et al., 2015), and DisGeNET (https://disgenet.org/) (Pinero et al., 2020) databases. The search condition was using the keywords “HBV hepatocellular carcinoma or hepatitis B Virus hepatocellular carcinoma or Hepatitis B Virus-Related Hepatocellular Carcinoma” and selecting the organisms “*Homo sapiens*”. We set the search terms to {[“HBV” (All Fields) AND “hepatocellular carcinoma” (All Fields)] AND “*H. sapiens*” (porgn)} AND “Expression profiling by array” (Filter), and found the proper microarray dataset GSE62232 in GEO database (https://ncbi.nlm.nih.gov/gds), which contained 10 HBV-related HCC and 10 normal liver samples (Schulze et al., 2015).

### Construction of co-expression network

WGCNA is a systems biology method that is used to correlate the modules to clinical traits through a soft-threshold algorithm, and Edmonson’s pathological grade was suitable clinical information for analysis (Qian et al., 2020). Using the R package of WGCNA in the SangerBox database (http://sangerbox.com/tool) (Langfelder and Horvath, 2008), we identified significant gene modules with the expression data in GSE62232. A weighted adjacency matrix was constructed according to a power function a_mn_ = |c_mn_|^*β*^ (c_mn_ = Pearson’s correlation between gene m and gene n; a_mn_ = adjacency between gene m and gene n). A scale-free co-expression network is constructed by the appropriate *β* value to increase the similarity matrix, and the adjacent matrix was transformed into a topological overlap matrix. The dynamic tree cut algorithm was used to detect gene modules. To identify hub modules, we set soft-thresholding power as 14 (scale-free *R* ^2^ = 0.85), and minimal module size as 20. The gene significance and module membership were respectively defined by the correlation coefficient of each module gene and each trait. In general, the higher the Pearson’s correlation coefficient relevant to the module the more important clinical significance.

### Identification of the key targets

The KXYA compound-putative targets and the hub module genes were mapped with the HBV-related HCC targets to obtain the overlapped genes (OGEs). Inputting the OGEs into the STRING database (https://string-db.org/) (Szklarczyk et al., 2019) with confidence scores ≥0.4 and the species limited to “*H. sapiens*”, then exported protein-protein interaction (PPI) data. The PPI data was analyzed by Cytoscape 3.8.0 (https://cytoscape.org/) (Kohl et al., 2011) and the non-connection genes were removed. We used the Analyzer plugin to analyze the PPI network and get the Degree, by taking over a double median of Degree to get the preliminary hub network (Zhang et al., 2014). Then, using CytoNCA plugin analyzes the preliminary hub network to get network topological parameters: Betweenness Connectivity (BC), Closeness Connectivity (CNC), and Degree Connectivity (DC) (Yu et al., 2019). We took the excess median of BC, CC, and DC to obtain a hub network. The MCODE application calculated the hub network to get the key module. The genes in key module from MCO are called key genes (Saito et al., 2012).

### Analysis of functional enrichment and survival

We performed respectively enrichment analyses of KXYA compound-putative targets and key genes by DAVID (https://david.ncifcrf.gov/) (Dennis et al., 2003). The functional enrichment analysis included the Gene Ontology (GO) and the Kyoto Encyclopedia of Gene and Genome (KEGG), and the GO analysis consists of Biological Process (BP), Molecular Function (MF), and Cellular Component (CC). The filtering of retrieval results is with a threshold value of *p* < 0.05 and counts in descending order. The main results have been shown as bubble plots. To evaluate the clinical prognostic significance of key genes in hepatitis-related HCC and HCC, we analyzed the correlation between the mRNA expression of key genes and the survival of patients in Kaplan Meier (http://kmplot.com/) (Menyhárt et al., 2018). The patients were divided into high expression group and low expression group, and log-rank *p*-values were used to evaluate the overall survival (OS) of the two groups.

### Exploration of the expression of the key genes

To explore the expression of key genes in HBV-related HCC, we verified the mRNA expression of key genes in the HBV-related HCC group versus the normal liver tissue group in the GSE62232 dataset, and outliers within the group were removed using a Box plot in SPSS 25.0 software (SPSS Inc., United States). Then we observed the expression of mRNA of key genes between HBV-related HCC tissues group and normal liver tissues group by The Cancer Genome Atlas (TCGA). To further explore the expression trends of key genes, the mRNA expression of key genes was verified in liver cancer data from TCGA and Oncomine (https://oncomine.org) (Rhodes et al., 2004) databases, and the protein situation of key genes was explored in HCC data from the HPA database (https://proteinatlas.org) (Uhlén et al., 2015).

### Quantitative real-time PCR

The 5 × 10^5^ HepAD38 cells were seeded in 6-well plates. After being incubated for 24 h, the cells were treated with KXYA (500 μg/ml) for 48 h. Total RNAs were extracted using the RaPure Total RNA Mini Kit (Magen, CN) according to the manufacturer’s instructions. The reverse transcription of total RNA to cDNA was performed with a qPCR RT Master Mix kit (TOYOBO, JAN). The quantitative Real-time PCR (qRT-PCR) was performed using the Real-time PCR Detection System (Agilent Technologies, United States) with the SYBR Green Real-time PCR Master Mix (TOYOBO, JAN). The primers used in this study are provided in Supplementary Table S3, using GAPDH as an internal control gene. The experiments were performed in triplicate and repeated 3 times.

### Statistical analysis

Data for graphing was processed with GraphPad Prism 8.0 software (GraphPad Software Inc., United States). Statistical analyses were performed using the SPSS 25.0 statistical software package. Data were expressed as the mean ± SD, two groups using student’s *t*-test and more than two groups using one-way ANOVA. Differences between groups are considered to be statistically significant if values of *p* < 0.05.

## Results

### KangXianYiAi formula inhibited the process of hepatitis B virus related hepatocellular carcinoma

**FIGURE 1:**
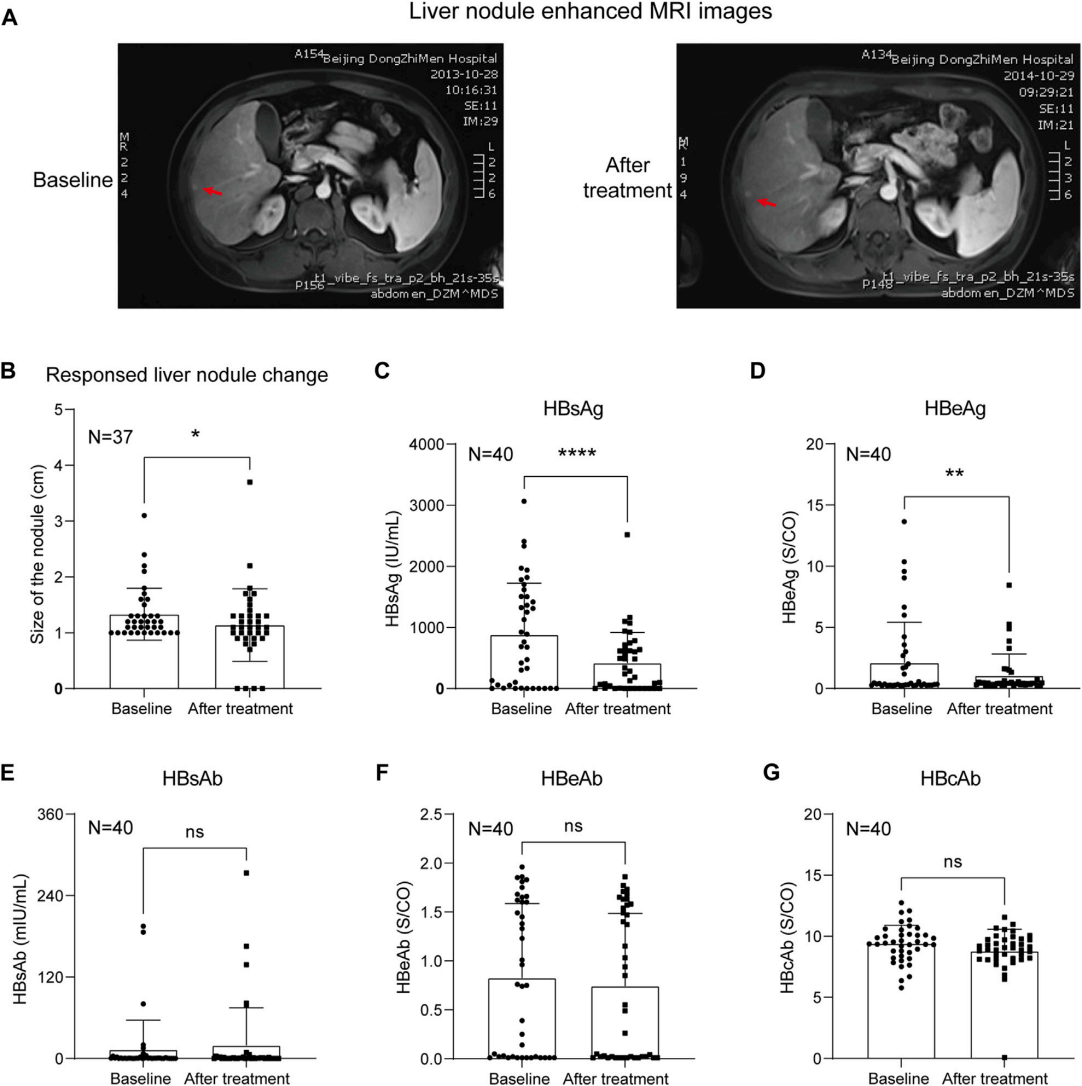
KXYA combined ETV treatment decreased the liver nodules of CHB patients. **(A)** The typical enhanced MRI image of the liver nodule in treatment case, red arrows pointed to the nodules. **(B)** Analysis of the liver nodules’ change in patients based on ultrasound. **(C)** Patients’ serological index HBsAg. **(D)** Patients’ serological index HBeAg. **(E)** Patients’ serological index HBsAb. **(F)** Patients’ serological index HBeAb. **(G)** Patients’ serological index HBcAb. Data are presented as the mean ± SD, *p*-value was calculated by performing paired Student’s *t*-test, *p < 0.05, **p < 0.01, ****p < 0.0001.

A total of 40 HBV-related liver precancerous patients whose serum HBV DNA levels were lower than the detection limit (less than 1 × 10^2^ IU/ml) and who had ultrasound or MRI exams before and after KXYA combined with ETV treatment were studied. And the enhanced MRI images of the typical case before and after KXYA treatment have shown in Figure 1A. Through the ultrasound test, the sizes of liver precancerous nodules before and after KXYA treatment were calculated. The result showed that after KXYA treatment, there were twenty-nine patients (72.5%) whose liver nodules became smaller or unaltered, eight (20%) patients’ liver nodules became larger, and there were three (7.5%) patients who had no response to treatment and processed to HCC. Except for patients who developed HCC, the liver precancerous nodules of the other patients after treatment with KXYA were significantly decreased (*p* < 0.05, Figure 1B). Meanwhile, we also analyzed the HBV serum indicators of the patients. The results showed that after treatment, the serum HBsAg (*p* < 0.0001, Figure 1C) and HBeAg (*p* < 0.01, Figure 1D) of these patients were significantly decreased, but there were not significant changes of serum HBsAb (Figure 1E), HBeAb (Figure 1F), and HBcAb (Figure 1G). The detailed information on HBV serum virology indicators were shown in Supplementary Table S4. These results indicated that KXYA could effectively inhibit the process of HBV-related HCC.

### KangXianYiAi formula suppressed proliferation and migration abilities of HepAD38 cells

**FIGURE 2:**
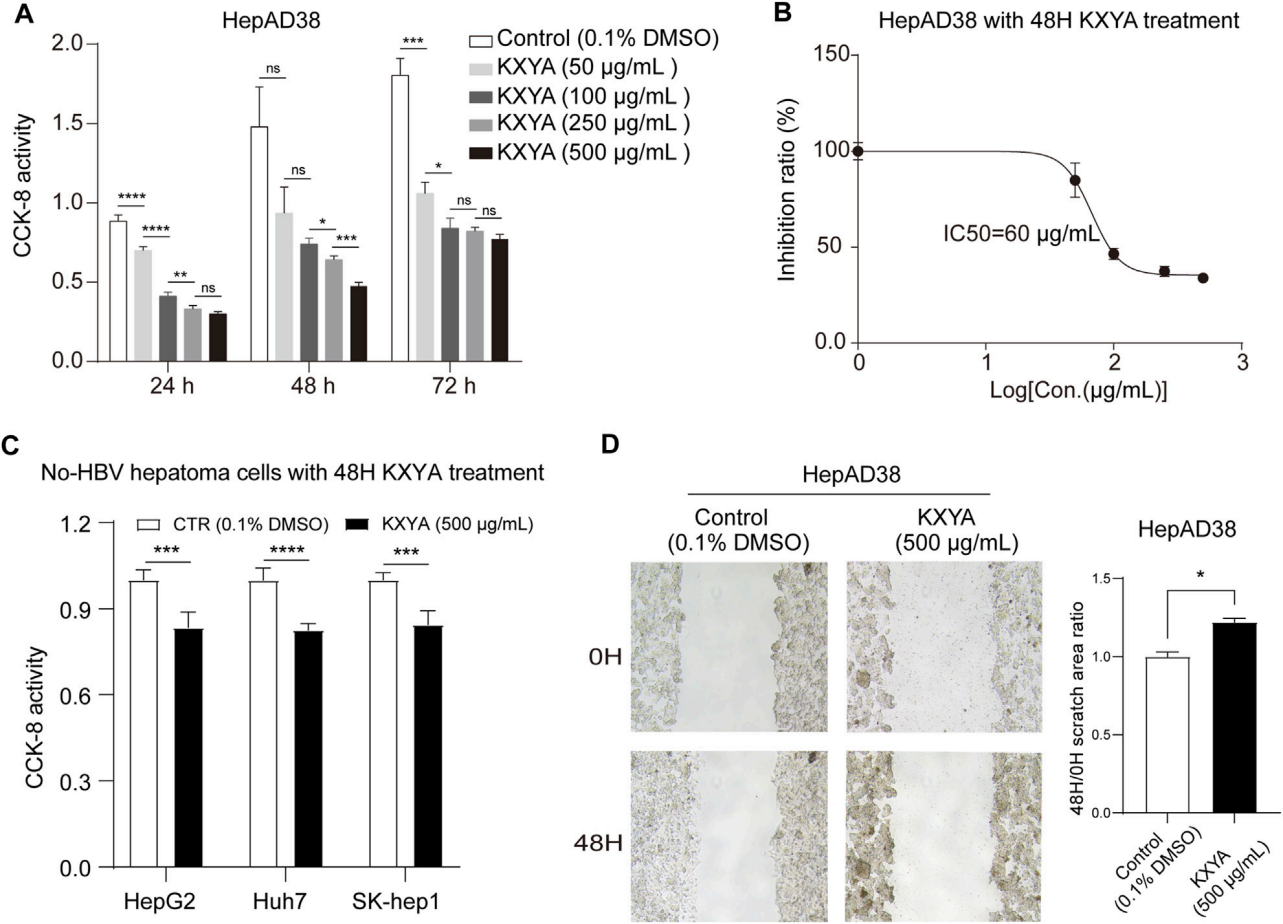
KXYA suppressed proliferation and migration of HepAD38 cells. **(A)** HepAD38 cells was treated with time- and dose-increased KXYA, and the cell viability was detected with CCK-8. **(B)** HepAD38 cells was treated with dose-increased concentration of KXYA for 48 h and the inhibitory concentration 50 (IC50) was calculated. **(C)** HepG2, Huh7, and SK-Hep1 cells were treated with KXYA (500 μg/ml) for 48 h, and the cell viability was detected with CCK-8. **(D)** The representative images and statistical analysis of migration assay for HepAD38 cells at 0 and 48 h with KXYA treatment. Data are presented as the mean ± SD, *p*-value was calculated by performing un-pair Student’s *t*-test, *, *p* < 0.05; **, *p* < 0.01; ***, *p* < 0.001, ****p < 0.0001; ns, not significant.

To confirm the inhibition effect of KXYA on HBV-related HCC, we treated HepAD38 cells with KXYA and detected the cell viability and migration activity. The CCK-8 result showed that, after treatment with different concentrations of the KXYA, the cell viability was significantly decreased in dose-dependent (Figure 2A), and the half-maximal inhibitory concentration (IC50) of KXYA with 48 h of treatment was 60 μg/ml (Figure 2B). Accordingly, for the no-HBV hepatoma cells including HepG2, Huh7, and SK-Hep1, KXYA also exhibited an inhibiting effect (Figure 2C). Meanwhile, the result of the cell wound scratch assay showed HepAD38 had a slower healing rate after KXYA treatment (Figure 2D). These results further supported the clinic curative effect of KXYA on HBV-related HCC *via* inhibiting cell proliferation and migration abilities, which might act by targeting both virus and cells.

### KangXianYiAi formula promoted apoptosis of HepAD38 cells *via* G2/M cell cycle arrest

**FIGURE 3:**
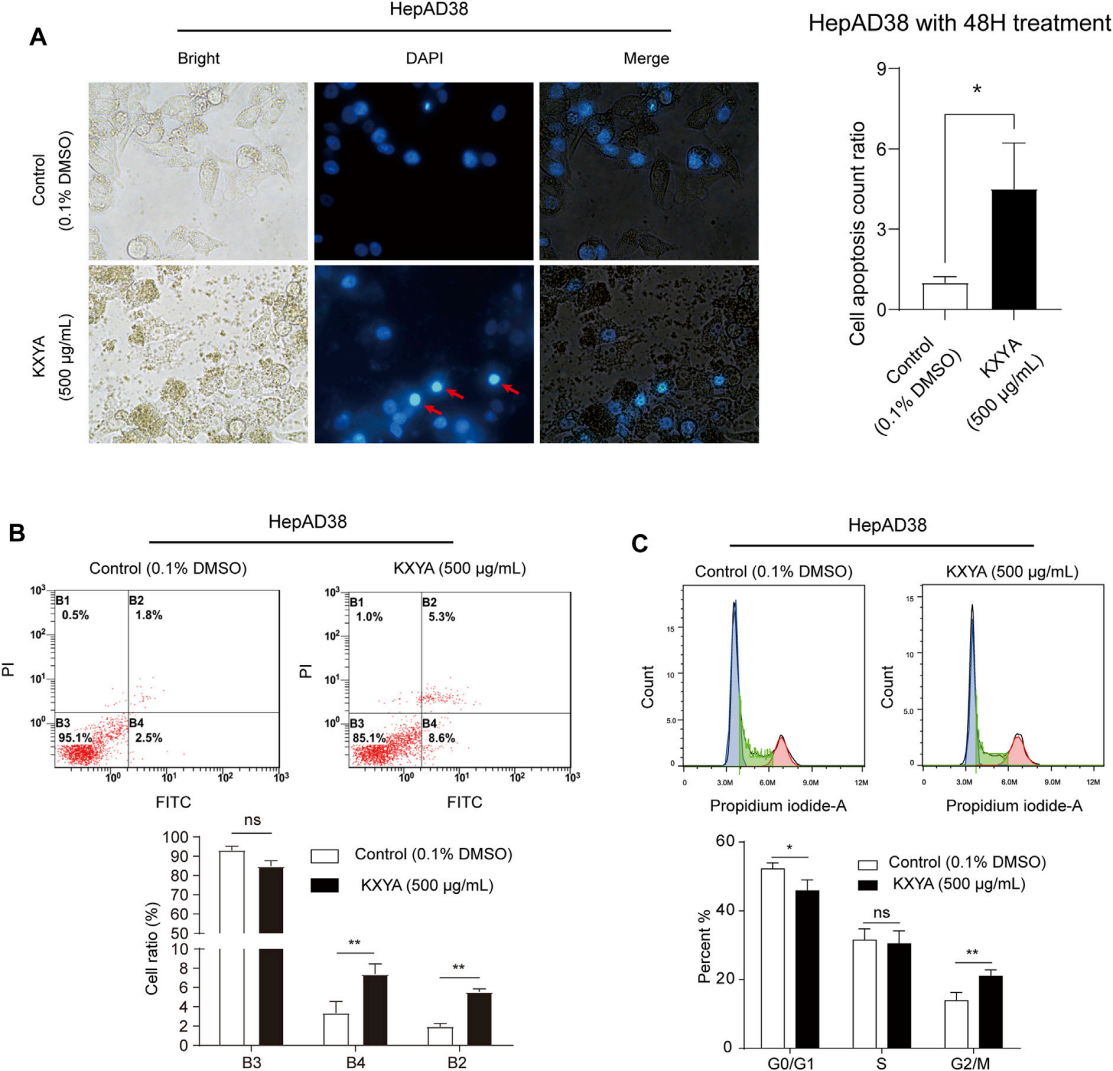
KXYA promoted apoptosis of HepAD38 cells. **(A)** HepAD38 cells was treated with KXYA (500 μg/ml) for 48 h, the representative images were DAPI fluorescence, bright and merge images, and statistical analysis of cell apoptosis count ratio. **(B)** HepAD38 cells was treated with KXYA (500 μg/ml) for 48 h, then performed PI/Annexin V-FITC fluorescent staining and flow cytometry. **(C)** HepAD38 cells was treated with KXYA (500 μg/ml) for 48 h, then performed PI fluorescent staining and flow cytometry. Data are presented as the mean ± SD, *p*-value was calculated by performing un-pair Student’s *t*-test, *, *p* < 0.05; **, *p* < 0.01; ns, not significant.

The cell proliferation decrease is usually caused by cell cycle arrest or cell death (Vermeulen et al., 2003). We found that KXYA treatment could result in a solidified nuclear morphology for cellular morphology and increased the cell apoptosis count ratio (Figure 3A). To confirm how KXYA functioned, we performed cell apoptosis and cycle tests. The flow cytometry test showed that the ratio of apoptotic and necrotic cells were both increased after KXYA treatment (Figure 3B). Furthermore, the cell cycle analysis result showed that the G0/G1 phase was decreased, the S phase was unaltered, and the G2/M phase was increased after KXYA treatment (Figure 3C). The G2/M cell cycle arrest always causes cell apoptosis. These results indicated that KXYA could lead to G2/M cell cycle arrest and cause HepAD38 cells apoptosis.

### The key targets of KangXianYiAi formula treatment on hepatitis B virus related hepatocellular carcinoma

**FIGURE 4:**
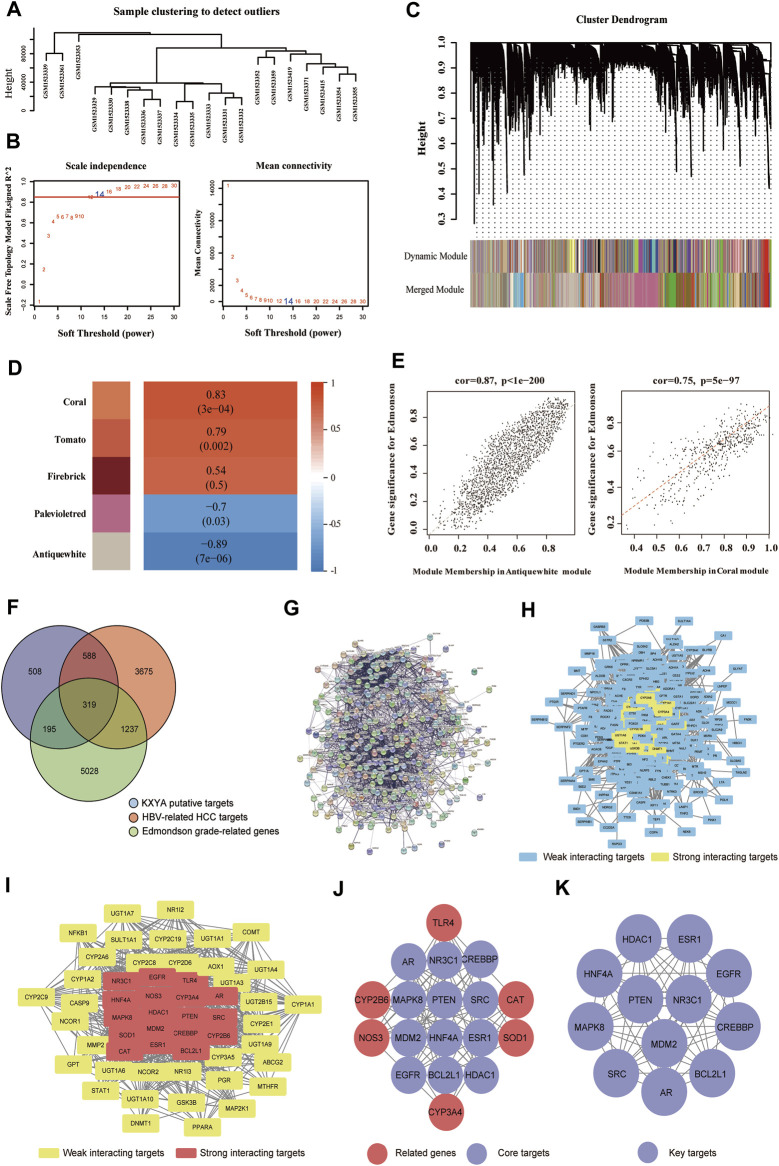
Obtained the key targets of KXYA on HBV-related HCC by network pharmacology analysis. **(A)** The sample clustering of the samples used in GSE62232 database. **(B)** The scale-free network analysis for samples in GSE62232 database. **(C)** The clustering dendrograms and modules for samples in GSE62232 database. **(D)** The heatmap of the top-5 modules, ranked by correlation between module genes and clinical traits of pathological grade. **(E)** The scatter plot of genes in the Coral and Antiquewhite modules. **(F)** The overlapped genes (OGEs) of KXYA treatment on HBV-related HCC, the blue circle represents KXYA putative targets, the red circle represents HBV-related HCC targets, and the green circle represents Edmonson grade-related genes. **(G)** The protein-protein interaction network of OGEs in STRING. **(H)** The protein-protein interaction network of OGEs in Cytoscape, the blue rectangles represent weak interacting target genes, the yellow rectangles represent strong interacting target genes. **(I)** The preliminary hub network of OGEs, the yellow rectangles represent weak interacting target genes, and the red rectangles represent strong interacting target genes. **(J)** The hub network of OGEs, the red circles represent related target genes, and the purple circles represent core target genes. **(K)** The Key module of OGEs, the purple circles represent key target genes. Pearson’s correlation test was used to calculate a correlation between two variable.

We firstly analyzed the four herbs in KXYA by CNKI and TCMSP databases, and the keywords were Chaihu, Huangqi, Shanyao, and Jiezi. The detailed information on all components in KXYA was in Supplementary Table S5. Next, according to the active components, we identified a total of 1,610 putative targets in KXYA by SwissTargetPrediction and STITCH analysis (Supplementary Table S6). We next analyzed HBV-related HCC disease targets, and subsequently obtained 5,819 disease targets of HBV-related HCC (Supplementary Table S7). To get the pathological grade-related genes of HBV-related HCC, we next used the GSE62232 dataset to perform a WGCNA analysis. There were 20 samplers in the sample clustering (Figure 4A), and we set a soft threshold power of *β* = 14 (scale-free *R* ^2^ = 0.85) to ensure a scale-free network (Figure 4B). After merging similar clusters, we identified modules that contained similar gene patterns (Figure 4C). From the top-5 heatmap of module-trait relationships (Figure 4D), we found that the 2 modules, Coral and Antiquewhite, were the most correlated with Edmonson’s pathological grade (Supplementary Table S8). Also, the gene significances for Edmonson in Coral and Antiquewhite modules were plotted, and the minuscule *p*-values indicated that they were highly correlated to the disease (Figure 4E). The Coral module positively correlated with Edmonson contained 745 genes, and the Antiquewhite module negatively correlated with Edmonson contained 6,034 genes, which were in Supplementary Table S9.

After taking the intersection of putative targets, we obtained 319 OGEs (Figure 4F) and constructed a PPI network of OGEs through the STRING database (Figure 4G). While, in the PPI network, there were 316 proteins imported into the Cytoscape to further obtain interaction networks (Figure 4H). Filtering with the network topological parameters, as Degree >30, we got a preliminary hub network of 54 interacting nodes (Figure 4I). With setting BC > 0.011, CC > 0.458, and DC > 39.5, we identified 18 highly connected nodes as the hub network (Figure 4J). We further analyzed the hub network with the MCODE plugin and finally obtained the key module with 12 genes, which were *AR, BCL2L1, CREBBP, EGFR, ESR1, HDAC1, HNF4A, MAPK8, MDM2, NR3C1, PTEN*, and *SRC* (Figure 4K).

### The signaling pathways involved in KangXianYiAi formula treatment on hepatitis B virus related hepatocellular carcinoma

**FIGURE 5:**
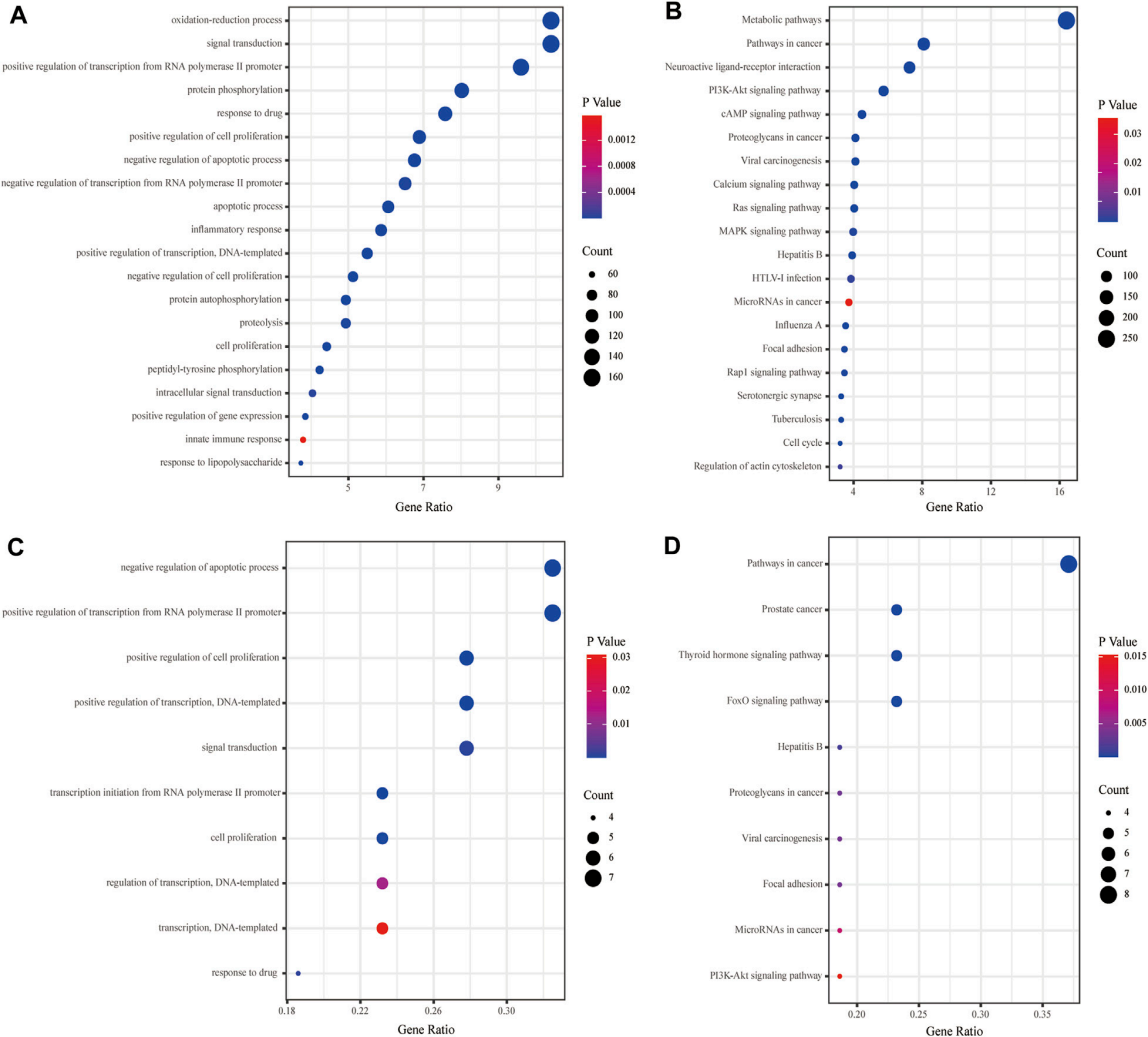
The functional enrichment analyses of the KXYA putative-target genes and the key genes. **(A)** Enriched biological processes of the KXYA putative-target genes. **(B)** Enriched KEGG pathways of the KXYA putative-target genes. **(C)** Enriched biological processes of the key genes. **(D)** Enriched KEGG pathways of the key genes.

The GO and KEGG analyses were performed for the putative targets and 12 key targets by the David database, and the specific information was in Supplementary Table S10. The GO enrichment analysis revealed that the GO-BP of KXYA putative target genes concentrated primarily on the oxidation-reduction process, signal transduction, and positive regulation of transcription from RNA polymerase II promoter (Figure 5A). And the KEGG enrichment analysis suggested that KXYA putative target genes were primarily associated with the metabolic, pathways in cancer and neuroactive ligand-receptor interaction (Figure 5B). To further explore the biological function, the key target genes were significantly related to multiple GO-BP, including negative regulation of the apoptotic process, positive regulation of transcription from RNA polymerase II promoter, and positive regulation of cell proliferation (Figure 5C). And the pathway enrichment results suggested that the key targets were mostly involved in pathways of cancer, prostate cancer, and thyroid hormone signaling pathway (Figure 5D). Based on the aforementioned results, both the putative targets and key targets of KXYA are enriched in pathways in cancer, hepatitis B, viral carcinogenesis, focal adhesion, microRNAs in cancer, proteoglycans in cancer, and *PI3K-Akt* signaling.

### The correlations between the key target genes expression and survival of hepatitis B virus related hepatocellular carcinoma

**FIGURE 6:**
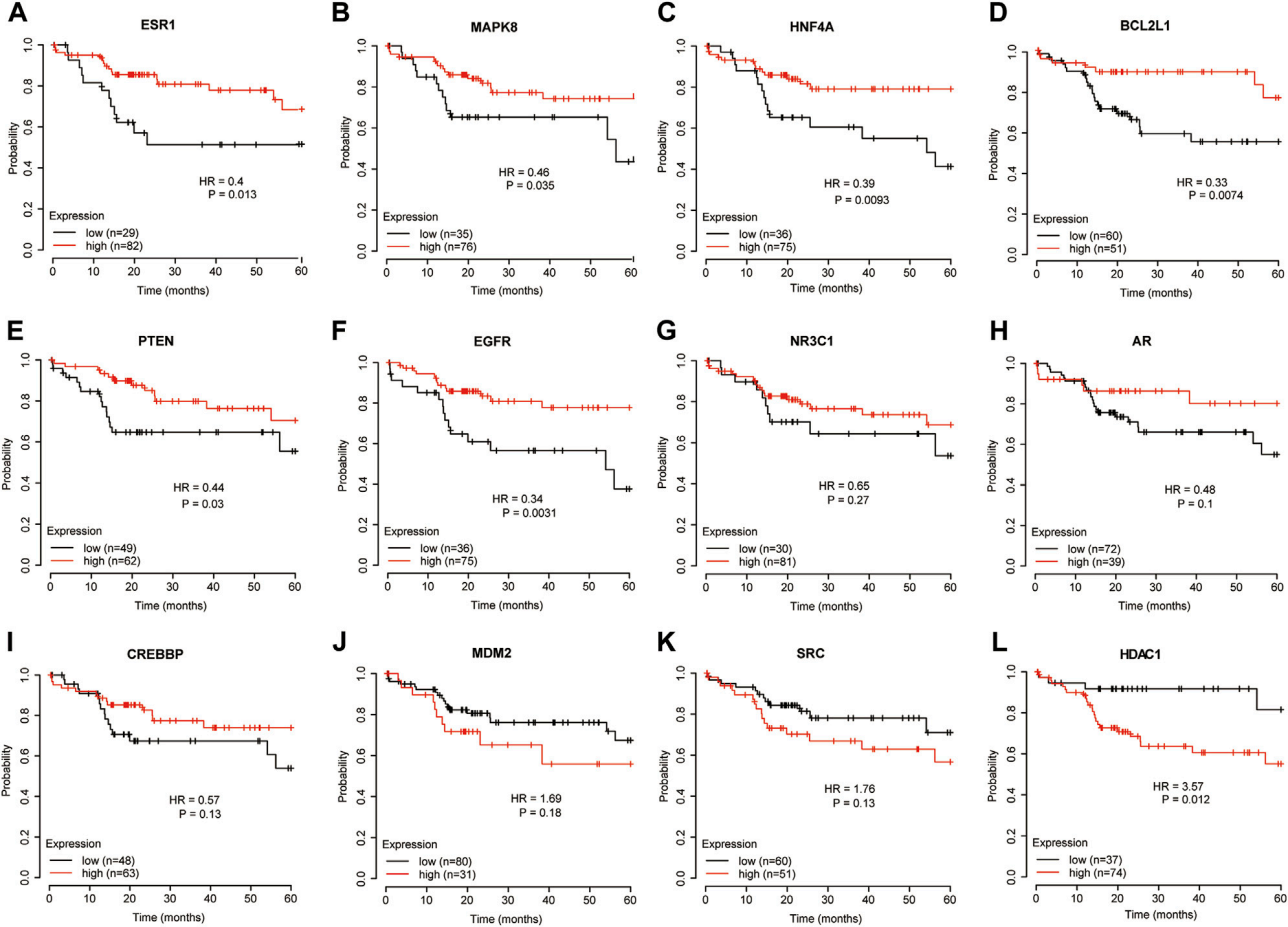
The survival prognostic analysis of the key genes in hepatitis-related HCC. **(A)** The overall survival analysis of ESR1. **(B)** The overall survival analysis of MAPK8. **(C)** The overall survival analysis of HNF4A. **(D)** The overall survival analysis of BCL2L1. **(E)** The overall survival analysis of PTEN. **(F)** The overall survival analysis of EGFR. **(G)** The overall survival analysis of NR3C1. **(H)** The overall survival analysis of AR. **(I)** The overall survival analysis of CREBBP. **(J)** The overall survival analysis of MDM2. **(K)** The overall survival analysis of SRC. **(L)** The overall survival analysis of HDAC1.The association of the key genes’ expression in tumor tissues and the prognosis of hepatitis-related HCC patients (*n* = 111) in TCGA database. Survival was calculated using Kaplan-Meier’s method and compared using log-rank test.

To further explore the correlations between the key genes and HBV-related HCC, we observed the relationship between key genes and prognosis in TCGA data. There were 364 patients with liver cancer in TCGA database, among which 111 patients had the risk factors of hepatitis virus and no alcohol consumption. When analyzing the overall survival of the two groups of patients, the basic information and risk factors of patients and the pathology of liver cancer were consistent. The survival analysis for the HCC patients with hepatitis risk factor were performed and the results showed that patients with higher expression of *ESR1, MAPK8, HNF4A, BCL2L1, PTEN*, and *EGFR* had a better 5-year overall survival (Figures 6A–F), while those with lower expression of *HDAC1* genes had a better prognosis (Figure 6L). Meanwhile, the prognosis of key genes in all TCGA HCC samples was also analyzed, and different from the previous analysis, the 5-year overall survival prognosis of HCC patients was better in the higher expression group of *AR, CREBBP, EGFR, ESR1, HNF4A, MDM2, NR3C1*, and *PTEN* (Supplementary Figures S2A–H), while the prognosis was better in the lower expression group of *HDAC1* and *SRC* (Supplementary Figures S2K,L). Overall, these results indicated that the expression of the key genes was related to HBV-related HCC patients’ survival and played an important role during hepatocarcinogenesis.

### The expression of the key target genes in liver tissues and HepAD38 cells with KangXianYiAi formula treatment

**FIGURE 7:**
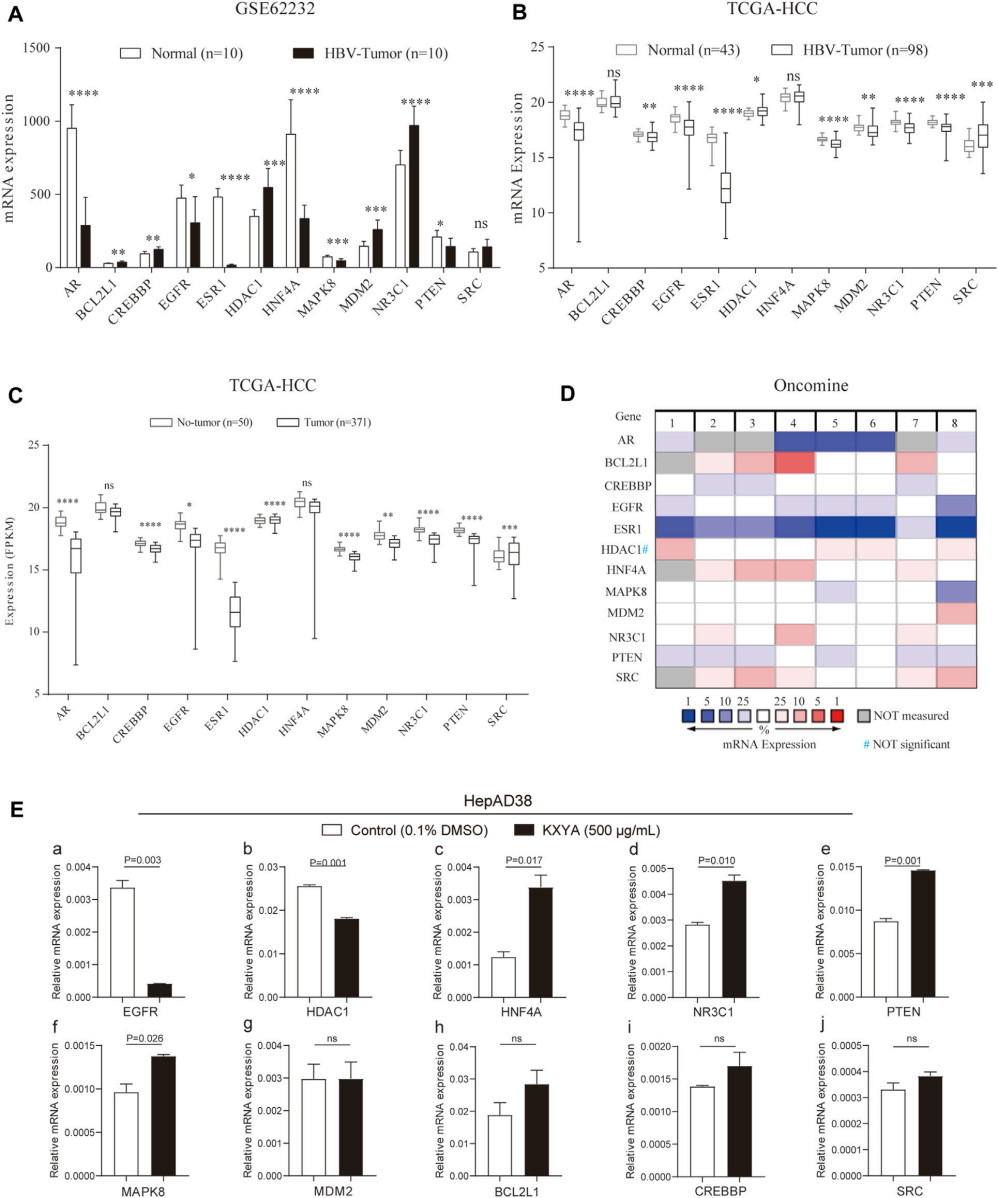
The mRNA expression of the key genes in public databases and HepAD38cells with KXYA treatment. **(A)** The mRNA expression of the key genes in GSE62232. **(B)** The mRNA expression of the key genes in HBV-related HCC of TCGA. **(C)** The mRNA expression of the key genes in HCC of TCGA. **(D)** The mRNA expression of the key genes in HCC of Oncomine. **(E)** HepAD38 cells was treated with KXYA (500 μg/ml) for 48 h and the key genes’ mRNA expression were detected by qRT-PCR, GAPDH was used as internal reference. Data are presented as the mean ± SD, *p*-value was calculated by performing un-pair Student’s *t*-test, ns, not significant.

We further analyzed the expression of the key genes in the public databases. In the GSE62232 dataset, the results showed that compared to normal liver tissues, B*CL2L1, CREBBP, HDAC1, MDM2*, and *NR3C1* genes were significantly upregulated in HBV-related HCC tissues, while *AR, EGFR, ESR1, HNF4A, MAPK8,* and *PTEN* genes were significantly downregulated (Figure 7A). In the TCGA database, compared to normal liver tissues, *HDAC1* and *SRC* were significantly upregulated in HBV-related HCC tissues, and *AR, CREBBP, EGFR, ESR1, MAPK8, MDM2, NR3C1,* and *PTEN* genes’ expression were significantly downregulated (Figure 7B), and the same results were observed between para-HCC and HCC tissues in all TCGA samples (Figure 7C). In the Oncomine database, we analyzed the expression of the key genes between normal liver tissues and HCC tissues, the results showed that among eight datasets, the expressions of *BCL2L1, HNF4A, MDM2, NR3C1*, and *SRC* were elevated in HCC tissues, while *AR, CREBBP, EGFR, ESR1, MAPK8,* and *PTEN* expressions were decreased (Figure 7D). The above analyses were performed at the mRNA level. At the protein level, we analyzed the expression of the key targets in the HPA database. The results of immunohistochemistry indicated that the protein expression of *AR, BCL2L1, CREBBP, EGFR, MAPK8*, and *PTEN* was downregulated (Supplementary Figures S3A–F), while *NR3C1, HNF4A,* and *HDAC1* were upregulated in the HCC sample compared to normal liver tissues (Supplementary Figures S3J–L).

Finally, to confirm the regulating effect of KXYA on target genes, we treated HepAD38 cells with KXYA (500 μg/ml) for 48 h and detected the target genes’ expression *via* qRT-PCR. And the results showed that among the target genes, *EGFR* and *HDAC1* were downregulated after KXYA treatment (Figures 7A,B,E), while *HNF4A, NR3C1, PTEN*, and *MAPK8* were upregulated (Figures 7C–F). These results indicated that the key targets’ expression alteration might play an important role during the HCC process. And during the treatment of KXYA on HBV-related HCC, expression of *EGFR, HDAC1, HNF4A, MAPK8, NR3C1*, and *PTEN* could be regulated, which were considered as further research targets.

## Discussion

HBV infection is one of the main reasons for hepatocarcinogenesis (Deng et al., 2016), and abnormalities of multiple genes are involved in the occurrence and progression of HBV-related HCC (Gómez-Moreno and Garaigorta, 2017). Currently, the treatment for HBV-related HCC is mainly the standardized treatment of HCC combined with anti-HBV therapy. However, due to the presence of virus reactivation, tumor recurrence, and other conditions, the clinic’s curative effect is not good enough (Li and Wang, 2016). In particular, for these low-level viremia CHB patients, even with ETV monotherapy, the incidence of HCC was still high, which suggested the importance of other more effective therapies (Kim et al., 2017; Yin et al., 2021). TCM is composed of a variety of compounds, with the characteristics of multi-target and holistic therapeutic effect (Zhou et al., 2014), which may be conducive to the treatment of HBV-related HCC. Here, we explored the function and mechanism of TCM formula KXYA on HBV-related HCC.

For hepatocarcinogenesis, there were several retrospective studies on liver nodules based on imaging have shown that the canceration rate of intrahepatic nodules without intervention was about 15%–35% after a median follow-up of 1 year (Motosugi et al., 2011; Kim et al., 2012; Hyodo et al., 2013). In our study, the canceration rate after the intervention of KXYA combined with ETV was 7.5% and there were 72.5% of patients had stable or smaller liver nodules. Furthermore, we found that KXYA could significantly inhibit the proliferation and migration abilities, and promote apoptosis of HepAD38 cells, and could also inhibit the cell viability of no-HBV hepatoma cells *in vitro* experiments. These results indicated that KXYA had a broad-spectrum effect on anti-HCC, and its inhibition effect on HBV-related HCC might be related to the regulation of the viability and apoptosis of HBV-infected or cancerous hepatocytes. Furthermore, the patients in our study have been treated with ETV monotherapy for a long time previously, which made the serum HBV DNA levels of patients before and after KXYA treatment all less than 100 IU/ml to detect. While other HBV serum indicators could be detected. And we found that HBsAg and HBeAg of patients were both decreased after KXYA treatment, while there was no significant change of HBsAb and HBeAb. The above results suggested KXYA might exert the function of accelerating HBV clearance by promoting the death of pathological cells infected with HBV. However, it was not clear whether KXYA has direct anti-HBV effects or functions on host immune activation, which needed further randomized controlled clinical trials, *in vitro* and *in vivo* experiments.

Meanwhile, the KXYA formula had a wide range of pharmacological targets, which made it is difficult to further investigate its efficacy mechanism in depth. Thus, we then used methods of network pharmacology and WGCNA to explore the mechanism of KXYA treatment on HBV-related HCC. We first constructed an interaction network between KXYA and HBV-related HCC, and after taking intersections with the targets of the disease and drugs, 12 key genes of KXYA treatment on HBV-related HCC were obtained, including *AR, BCL2L1, CREBBP, EGFR, ESR1, HDAC1, HNF4A, MAPK8, MDM2, NR3C1, PTEN,* and *SRC*. To further explore the expression and function of these key genes in HBV-related HCC, we performed GO and KEGG analysis and the results showed that the biological functions of potential targets and key genes of KXYA were mainly associated with pathways in cancer, hepatitis B, viral carcinogenesis, focal adhesion, microRNAs in cancer, proteoglycans in cancer, and *PI3K-Akt* signaling, which indicated the closed correlation between the key genes to HBV-related HCC. We next analyzed the expression of key genes in GSE62232 and TCGA databases, and the results showed that compared with normal liver tissues, the expression of *AR, EGFR, ESR1, MAPK8,* and *PTEN* were downregulated in HBV-related HCC tissues, and *HDAC1* expression was upregulated. Meanwhile, we observed that in hepatitis-related HCC, patients with higher expression of *BCL2L1, EGFR, ESR1, HNF4A, MAPK8,* and *PTEN*, and lower expression of *HDAC1* had a better clinical prognosis. These results indicated that the key genes we obtained might play an important role in HBV-related HCC.

Additionally, according to previous reports, most of the key genes we obtained exert certain anti- or promoting HCC functions. In HBV-related HCC, the decreased expression of *HNF4A* enhances potential oncogenic GALNT10 protein activity (Wu et al., 2015). In the early stages of HCC, *HNF4A* could inhibit cell proliferation of HCC through the miR-122-adam17 pathway (Yang et al., 2020), and downregulation of *HNF4A* could lead to loss of hepatocyte characteristics (Ramesh and Ganesan, 2016). The higher expression of *MAPK8* is correlated to the lower tumor recurrence for HCC patients (Gao et al., 2018), and upregulation of *MAPK8* could inhibit the proliferation, migration, and invasion abilities of mouse HCC cell lines (Zhang et al., 2016). *NR3C1* encodes glucocorticoid receptors and is considered to be a tumor suppressor gene, which controls cell proliferation, differentiation, and apoptosis (Matthews et al., 2015). *PTEN* plays a critical role in antiviral innate immunity and the development of HCC, and HBV infection could downregulate the expression of *PTEN via* increasing N6 methyladenosine modification of *PTEN* RNA, which leads to its degradation (Kim et al., 2020). The HBx promotes cell proliferation by disturbing the cross-talk between *PTEN* and miR-181a in HBV-related hepato-carcinogenesis (Tian et al., 2017). *EGFR* (Crouchet et al., 2022) and *HDAC1* (Rivas et al., 2021) have a role in promoting hepatocarcinogenesis. The upregulation of *EGFR* promotes the progression of HCC and leads to sorafenib resistance (Pang et al., 2019). *HDAC1* expression is correlated with downregulation of *PTEN*, and poor prognosis of HCC patients (Wang et al., 2017).

*PTEN*, *HNF4A*, *HDAC1*, *EGFR,* and *MAPK8* were involved in proliferation, migration, and apoptosis in HCC. And by qRT-PCR assay, we found that KXYA treatment could upregulate the expression of *HNF4A*, *MAPK8*, *NR3C1,* and *PTEN*, and downregulated the expression of *EGFR* and *HDAC1* in HepAD38 cells. Through previous research and our experimental results, KXYA could induce cell apoptosis by increasing the mRNA expression of *PTEN* and *HNF4A*, thereby inhibiting the proliferation of HBV related hepatoma cell lines. To conclude, these key genes may be potential targets for HBV-related HCC pathogenesis and therapy of KXYA. However, we need to further explore the expression and function of the key genes with KXYA treatment *in vivo* with clinic samples and animal models.

## Conclusion

In summary, our results demonstrated that the KXYA formula could exert the treatment effect on HBV-related HCC by regulating hepatocyte proliferation, migration, and apoptosis. During the treatment, the expression of *HNF4A, MAPK8, NR3C1, PTEN, EGFR*, and *HDAC1* was regulated by KXYA, indicating their important roles during the tumorigenesis and progression of HBV-related HCC and might be the targets for the clinical diagnosis and therapy.

## Data Availability

The datasets analyzed during the current study are available from the corresponding author on reasonable request. All the relevant data is provided within the paper and its supporting information files.

## Glossary

HCC: Hepatocellular carcinoma
HBV: hepatitis B virus
CHB: chronic hepatitis B
cccDNA: covalently closed circular DNA
TCM: traditional Chinese medicine
KXYA: KangXianYiAi
WGCNA: weighted gene co-expression network analysis
ETV: Entecavir
DMEM: dulbecco’s modified eagle medium
FBS: fetal bovine serum
DMSO: dimethylsulfoxide
PI: propidium iodide
OB: oral bioavailability
DL: drug-likeness
OGEs: overlapped genes
PPI: protein-protein interaction
BC: Betweenness Connectivity
CNC: Closeness Connectivity
DC: Degree Connectivity
GO: Gene Ontology
KEGG: Kyoto Encyclopedia of Gene and Genome
BP: Biological Process
MF: Molecular Function
CC: Cellular Component
OS: overall survival
TCGA: The Cancer Genome Atlas
qRT-PCR: Quantitative Real-time PCR
AR: Androgen receptor
BCL2L1: BCL2 like 1
CREBBP: CREB binding protein
EGFR: Epidermal growth factor receptor
ESR1: Estrogen receptor 1
HDAC1: Histone deacetylase 1
HNF4A: Hepatocyte nuclear factor 4 alpha
MAPK8: Mitogen-activated protein kinase 8
MDM2: MDM2 proto-oncogene
NR3C1: Nuclear receptor subfamily 3 group C member 1
PTEN: phosphatase and tensin homolog
SRC: SRC proto-oncogene, non-receptor tyrosine kinase
