# Supplementary material for: Network pharmacology and in vitro experiments-based strategy to investigate the mechanisms of KangXianYiAi formula for hepatitis B virus-related hepatocellular carcinoma
Source: Front Pharmacol. 2022 Sep 5;13:985084. doi: 10.3389/fphar.2022.985084 (PMC9483169; doi:10.3389/fphar.2022.985084)
Supplement: Supplementary file 2 [file Image1.pdf]

Network pharmacology and in vitro experiments-based strategy to investigate the mechanisms of KangXianYiAi formula for hepatitis B virus-related hepatocellular carcinoma

Xu Cao[1#], Hening Chen[1#], Zhiguo Li [2#], Xiaoke Li[1,3], Xianzhao Yang[1,3], Qiushuo Jin[1,4], Yijun Liang[1], Jiaxin Zhang[1], Meiyue Zhou[1], Ningyi Zhang[1], Guang Chen[1,3*], Hongbo Du[1, 3*], Xiaobin Zao[1, 4*], Yong'an Ye[1, 3*]

[1] Dongzhimen Hospital, Beijing University of Chinese Medicine, 100700 Beijing, China;

[2] Beijing Fengtai Hospital of Integrated Traditional and Western Medicine, 100072 Beijing, China;

[3] Institute of Liver Diseases, Beijing University of Chinese Medicine, 100700 Beijing, China;

[4] Key Laboratory of Chinese Internal Medicine of Ministry of Education and Beijing, Dongzhimen Hospital, Beijing University of Chinese Medicine, 100700 Beijing, China.

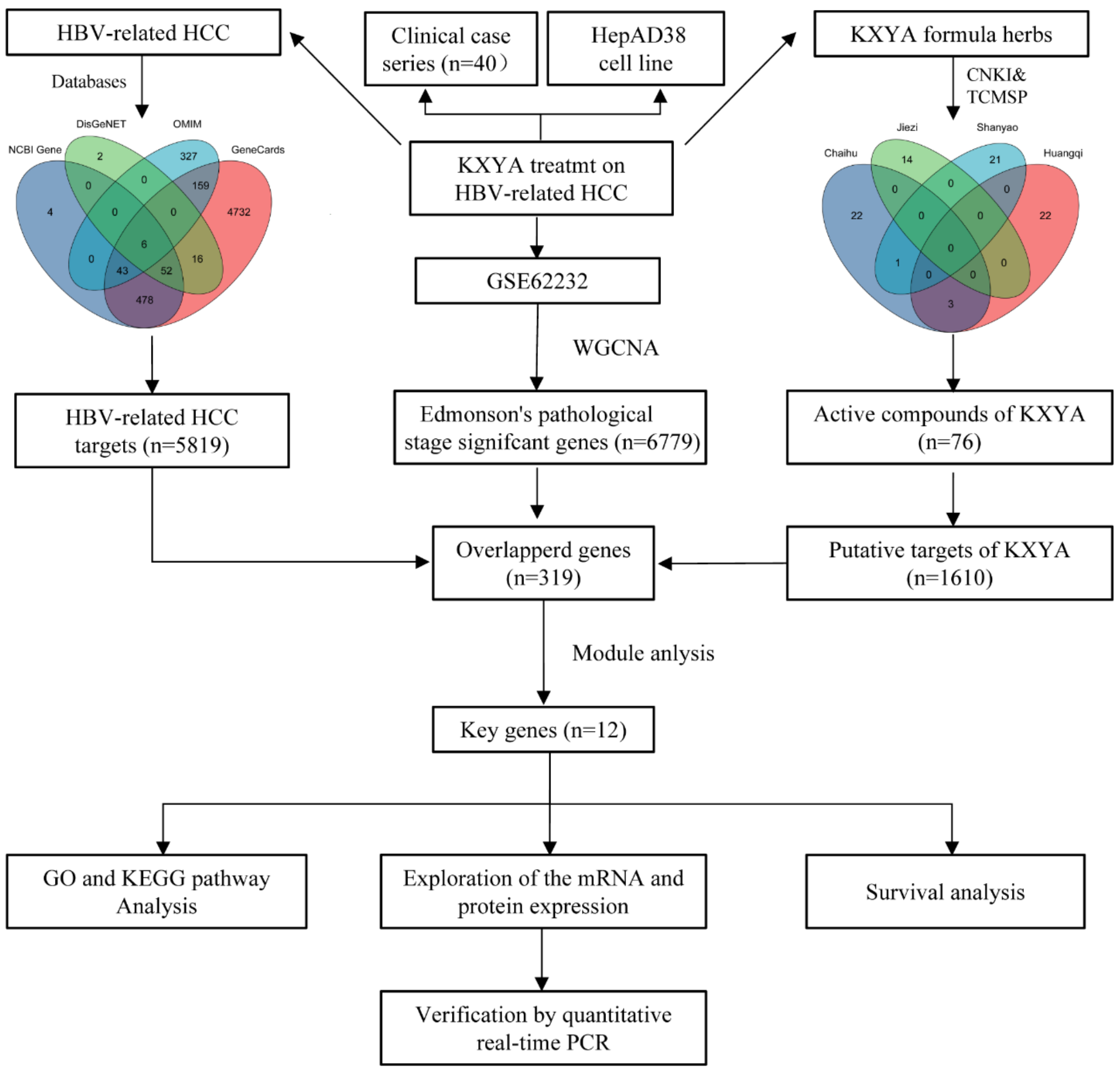

Figure S1. Flowchart of the study. To explore the efficacy and mechanism of KXYA, we first analyzed the clinical case series treated by KXYA, and performed cell experiments with KXYA treatment on HepAD38. Next, we used network pharmacology analysis combined with WGCNA to obtain the key genes, and the GO and KEGG pathways enrichment analysis, prognostic value and expression levels of the key genes were evaluated with multiple databases. Finally, qRT-PCR was applied to verify the regulation effect of KXYA on the key genes.

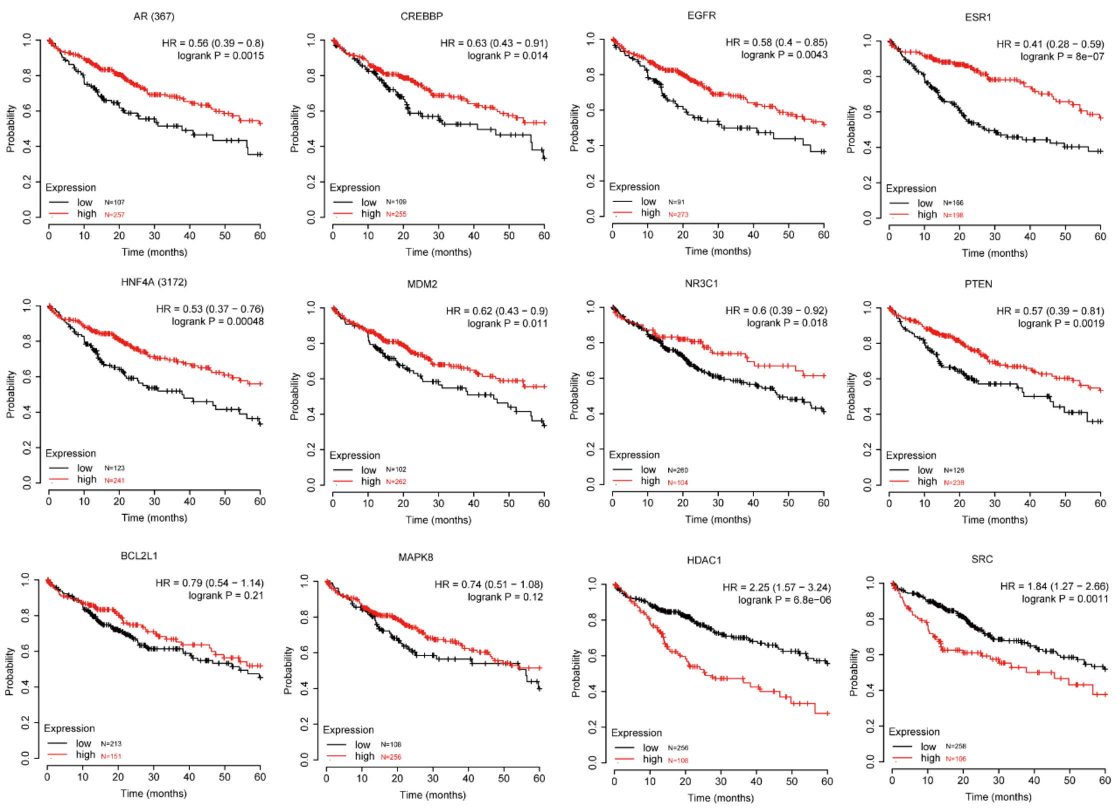

Figure S2. The survival prognostic analysis of the key genes in HCC. A-L. The association of the key genes' expression in tumor tissues and the prognosis of HCC patients (n=364) in TCGA database. Survival was calculated using Kaplan-Meier's method and compared using log-rank test.

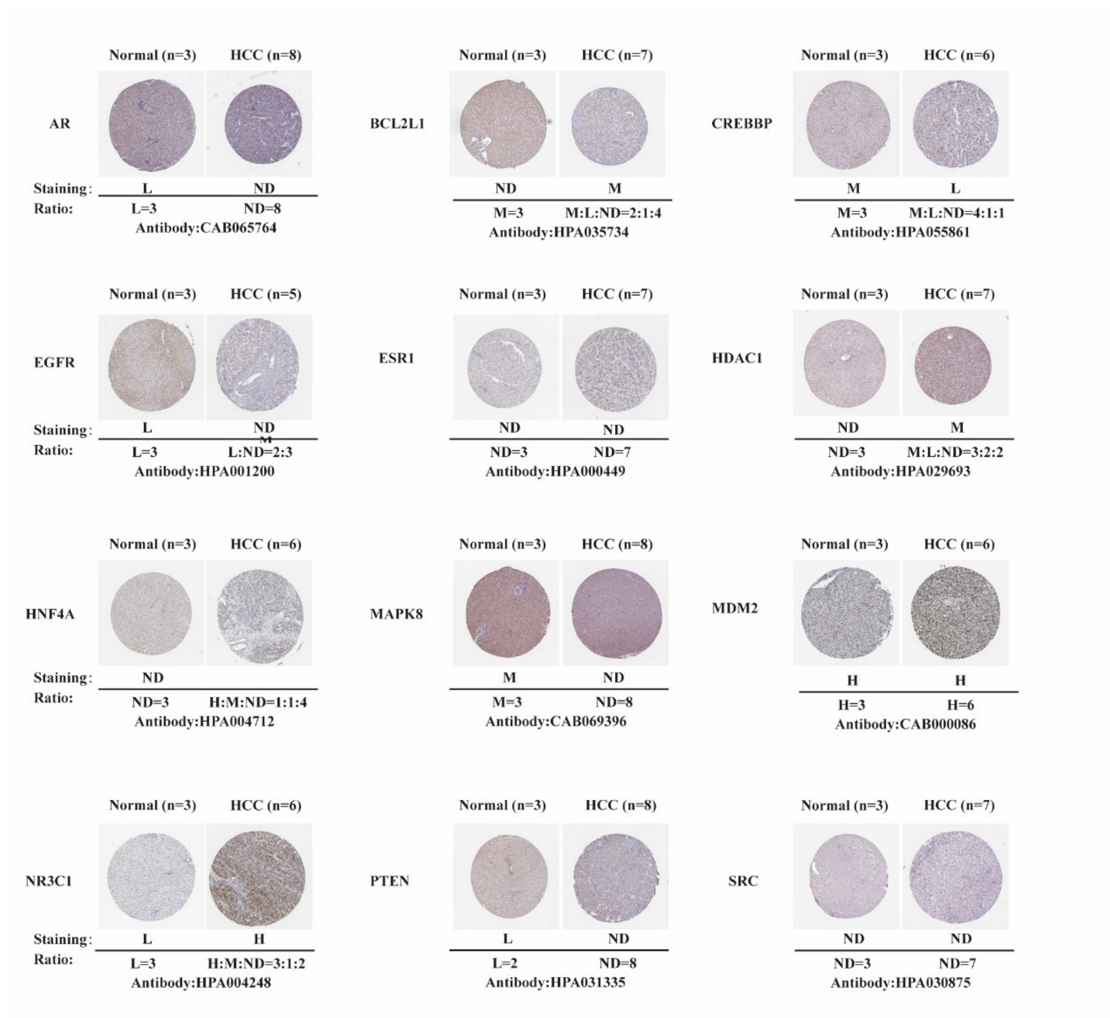

Figure S3. The protein expression of the key genes. A-L. The representative immunohistochemistry images of the key genes in HCC tissues and normal liver tissue based on Human Protein Atlas.
